# Effects of Trophic Skewing of Species Richness on Ecosystem Functioning in a Diverse Marine Community

**DOI:** 10.1371/journal.pone.0036196

**Published:** 2012-05-31

**Authors:** Pamela L. Reynolds, John F. Bruno

**Affiliations:** 1 Department of Biology, University of North Carolina at Chapel Hill, Chapel Hill, North Carolina, United States of America; 2 Department of Biological Sciences, Virginia Institute of Marine Science, The College of William and Mary, Gloucester Point, Virginia, United States of America; Umea University, Sweden

## Abstract

Widespread overharvesting of top consumers of the world’s ecosystems has “skewed” food webs, in terms of biomass and species richness, towards a generally greater domination at lower trophic levels. This skewing is exacerbated in locations where exotic species are predominantly low-trophic level consumers such as benthic macrophytes, detritivores, and filter feeders. However, in some systems where numerous exotic predators have been added, sometimes purposefully as in many freshwater systems, food webs are skewed in the opposite direction toward consumer dominance. Little is known about how such modifications to food web topology, e.g., changes in the ratio of predator to prey species richness, affect ecosystem functioning. We experimentally measured the effects of trophic skew on production in an estuarine food web by manipulating ratios of species richness across three trophic levels in experimental mesocosms. After 24 days, increasing macroalgal richness promoted both plant biomass and grazer abundance, although the positive effect on plant biomass disappeared in the presence of grazers. The strongest trophic cascade on the experimentally stocked macroalgae emerged in communities with a greater ratio of prey to predator richness (bottom-rich food webs), while stronger cascades on the accumulation of naturally colonizing algae (primarily microalgae with some early successional macroalgae that recruited and grew in the mesocosms) generally emerged in communities with greater predator to prey richness (the more top-rich food webs). These results suggest that trophic skewing of species richness and overall changes in food web topology can influence marine community structure and food web dynamics in complex ways, emphasizing the need for multitrophic approaches to understand the consequences of marine extinctions and invasions.

## Introduction

Species losses from habitat destruction and overharvesting, and species gains from accidental and intentional introductions, are changing the topology of food webs with consequences for ecosystem functioning [1], [2]. Despite ongoing losses of species richness at the global scale [3], the rate of species gain from introductions at local scales can outpace those lost to extinctions with potentially little effect on overall community diversity [4], [5]. However, inherent species biases in extinction and invasion processes are altering the *distribution* of diversity across trophic levels [2], [6] with potential effects on ecosystems [7]. While natural food webs are thought to be slightly weighted toward greater species richness at lower trophic levels [8], biases in which species are more likely to be lost and gained can result in food webs skewed toward ratios of greater or lower predator to prey richness.

Local species extirpations are often biased toward species at higher trophic levels. Large consumers such as top predators are generally more likely to go extinct due to their characteristic small population sizes, small geographic ranges, slow population growth, and high susceptibility to over-harvesting and habitat loss [9], [10], [11], [12], [13]. However, recent analyses on global fisheries stock assessments and landings indicate that small-bodied consumer populations are also highly susceptible to collapse, with substantial impacts on adjacent trophic levels in oceanic food webs [14].

Despite documented global declines in consumer guilds [15], there are many examples of local increases in predator richness due to species introductions and range shifts, such as those observed in fishes, snakes and birds in the Everglades [16], [17], lionfish in the Caribbean [18], [19], and seagrass-associated fishes in the Gulf of Mexico [20]. Intentional or accidental releases of predatory game fish in streams and lakes modified for human recreation have also elevated local predator richness in North American and European waterways [21], [22]. From brown tree snakes in Guam, Burmese pythons in the Everglades, Asian carp in the Great Lakes, cactus moths in Mexico, cane toads in Australia, and black rats and feral cats around the world, many striking examples of the negative impacts of invasive species come from the establishment of exotic consumers [23], [24]. However, in general localized species gains may be inherently biased toward species at lower trophic levels due to mechanisms of transport and establishment. For example, in estuarine systems species at lower trophic levels, such as macroplanktivores and plants (particularly species found in ballast water), are more likely to be transported and become established [6]. Highly evolved dispersal and hitchhiking abilities, as well as traits such as fast growth and reproduction of plants, fungi, and invertebrates may promote their transport as well as spread and establishment in new regions [25].

Regardless of the direction, trophic skew (a re-organization of trophic structure due to a change in the ratio of predator to prey richness [2], [26]) is changing the structural biodiversity of natural food webs with unknown consequences for ecosystem processes [6], [8], [27]. However, understanding the consequences of shifts in species richness across multiple trophic levels is difficult because diversity effects are often contingent upon the presence and diversity of adjacent trophic levels [28], [29]. Capture and consumption of prey from within a given trophic level can be influenced by richness at lower and higher trophic levels [30], and diversity can affect production through fundamentally different mechanisms across trophic levels [31]. Thus explicit investigation of how species gains and losses, both consequences and drivers of global change, simultaneously affect ecosystem functioning is key to understanding current and future responses of natural ecosystems to trophic skew [7].

While a wealth of research over the past decade indicates that changes in biodiversity alter ecosystem functioning and services [32], [33], [34], most studies have focused on manipulating richness at only one or, more recently and rarely, two trophic levels [35]. Manipulations of species richness across multiple trophic levels typically employ designs in which overall community richness varies across treatments [36], and/or feature limited species pools where results are largely ascribed to identity effects and changes in community composition [37]. Because elevated system diversity is known to affect production and other ecosystem properties [38], it is often difficult to partition the effects of changes in predator to prey richness from overall changes in diversity at the community level. Expect perhaps work by Srivastava et al. [39], to date, no study has explicitly tested the effects of trophic skew on specific ecosystem properties including the strength of a trophic cascade.

**Figure 1:**
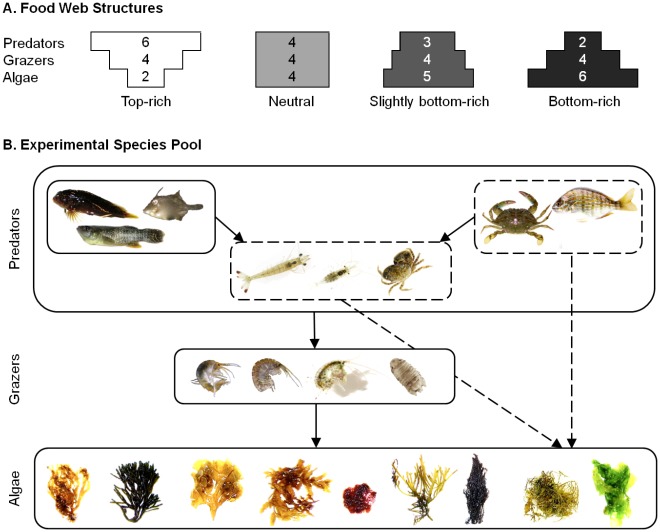
Experimental food webs. A) Structure of experimental food webs with varying species richness per trophic level, i.e., trophic skew, and B) experimental species pool (predators: *Hypleurochilus geminatus, Monacanthus hispidus, Fundulus heteroclitus*, swimming crabs (*Callinectes sapidus* or *C. similis*), *Lagodon rhomboids, Penaeus aztecus, Palaemonetes vulgaris*, mud crabs (*Panopeus herbstii, Eurypanopeus depressus* or *Neopanope sayi*); grazers: *Gammarus mucronatus, Elasmopus levis, Dulichiella appendiculata, Paracerceis caudata*; macroalgae: *Dictyota menstrualis, Codium fragile, Padina gymnospora, Sargassum filipendula, Ceramium sp., Gracilaria tikvahiae, G. verrucosa, Hypnea musciformis, Ulva lactuca*. Species are not drawn to scale.

We empirically tested how trophic skew can influence ecosystem functioning in a diverse tri-trophic estuarine food web in outdoor mesocosms by simultaneously manipulating plant and predator species richness while holding overall community richness constant. Using a diverse species pool, we created four food web structures (Fig. 1A) that reflected real or predicted degrees of trophic skewing of natural systems: 1) top-rich food webs (inverted triangle shape) with greater ratios of predator to prey richness, consistent with predictions based on accidental and intentional predator additions [40], 2) neutral (rectangular shape) with constant predator to prey richness ratios, and 3–4) two degrees of bottom-rich food webs (triangular shapes) with greater ratios of prey to predator richness, as may be typical for impacted estuarine food webs [6]. We tested the direct and indirect effects of predator (top-rich) and plant (bottom-rich) diversity [41], [42], [43] by comparing production and trophic cascade strength across these trophic skewing scenarios. With concurrent and opposite changes in prey and predator richness, it is possible that 1) the species richness at one trophic level may dominate and dictate final primary producer biomass, 2) effects of concurrent changes in richness may cancel out, resulting in constant primary production across different food web structures, or 3) the concurrent changes in richness may interact additively or nonadditively [30], [44].

Based on known dynamics in this and similar experimental systems where predator richness promoted predation and increased top-down control of grazer populations [44], [45], we expected that top-rich food webs (greater predator to prey richness) would exhibit lower grazer abundances and higher algal biomass compared to bottom-rich food webs (lower predator to prey richness). However, algal richness is known to promote primary as well as secondary production [37], [46], and thus bottom-up food webs could have either higher or lower algal biomass depending on the intensity of grazing. If predator (top-down) richness effects drive this system, we expected top-rich food webs to have a stronger positive trophic cascade on algal biomass compared to bottom-rich food webs, and the reverse if algal (bottom-up) richness is more important.

## Results

**Figure 2:**
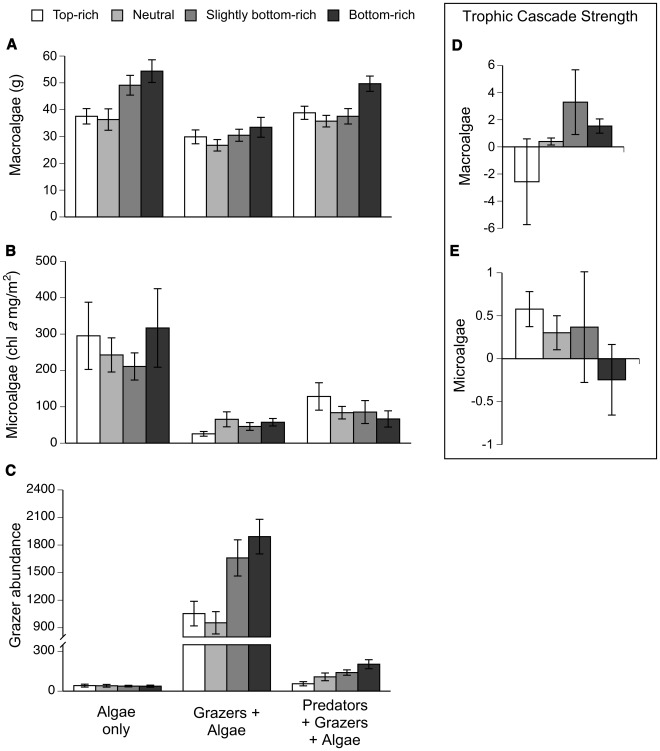
Algal and grazer responses to trophic skew. Final A) biomass of experimentally stocked macroalgae, B) colonizing algae (e.g., microalgae) chlorophyll *a* accumulation C) mesograzer abundance, and the trophic cascade strength on macroalgae (D) and microalgae (E) in experimental mesocosms across trophic skewing treatments after 24 days. Higher values in D and E indicate a stronger positive trophic cascade on the algae. Values are means±1SE.

The presence of consumers and the distribution of species richness across trophic levels (trophic skew) influenced final algal biomass and grazer abundance (Fig. 2, Table 1). Generally, grazers reduced the wet mass of experimentally stocked macroalgae (hereafter referred to as macroalgae) by 33% compared to grazer-free controls (LSM contrast, F_1,83_ = 42.64, P<0.001), but this effect disappeared when their predators were present, indicating a trophic cascade (Fig. 2A; Table 1; see Fig. S2 for images of final macroalgal biomass). Predators generally increased macroalgal biomass (LSM contrast, F_1,83_ = 10.44, P<0.002). The strongest positive trophic cascade on macroalgae appeared in the bottom-rich food webs (e.g., triangular shaped, Fig. 2D).

**Table 1:** Results of two-factor anova on the effects of trophic skew and food chain length.

Response Factor	d.f.	SS	*F*	*P*
***Macroalgal biomass***				
Food chain length (FCL)	2	3875.99	26.59	**<0.001**
Trophic skew (TS)	3	2589.50	11.84	<**0.001**
FCL * TS	6	555.11	1.27	0.281
Error	83	6050.01		
***Microalgal accumulation***				
Food chain length	2	10.17	40.08	**<0.001**
Trophic skew (TS)	3	0.11	0.28	0.841
FCL * TS	6	1.44	1.89	0.092
Error	83	10.53		
***Grazer abundance***				
Food chain length	1	10703.58	440.05	**<0.001**
Trophic skew (TS)	3	958.84	13.14	**<0.001**
FCL * TS	3	178.40	2.44	0.073
Error	56	1362.12		

The degree of trophic skewing encompassed the presence of upper trophic levels (i.e., food chain length: algae only,+grazers,+grazers+predators) and the distribution of species richness (top-rich, neutral, slightly bottom-rich, bottom-rich skewed) on final A) macroalgal biomass, B) microalgal accumulation, and C) grazer abundance. Only data from replicates in which grazers were initially stocked (all treatments except ‘algae only’) were used in the analysis of food chain length and richness distribution treatment effects on grazer abundance (C).

Microalgae (primarily the chain-forming diatom *Tabellaria sp.)* and some early-successional *Cladophora sp*. and *Ulva linza* colonized and grew in all mesocosms. Chlorophyll *a* concentration, a proxy for growth of these naturally recruiting algae (hereafter referred to as “microalgae” due to their small size and dominance by diatoms), was affected by the presence of upper trophic levels (food chain length treatment) but not by trophic skew (Fig. 2B, Table 1). On average, grazers reduced chlorophyll *a* concentration by 83% in the absence of predators, and 66% in their presence (Fig. 2B). Predators generally promoted microalgal accumulation (LSM contrast, F_1,83_ = 4.19, P = 0.044). In contrast with the macroalgal results, the strongest trophic cascade on microalgal accumulation emerged in the top-rich food webs (inverted triangle) with a greater ratio of predator to prey species richness, although this response was highly variable (Fig. 2E).

**Figure 3:**
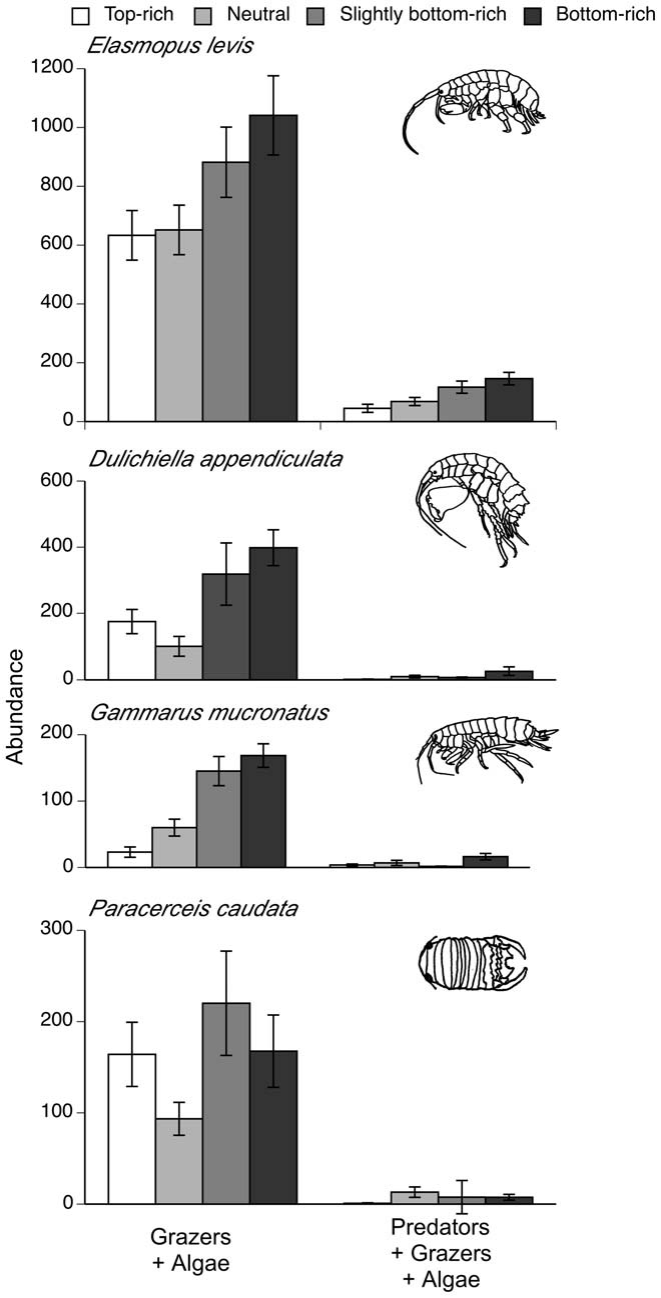
Grazer species responses to trophic skew. Final grazer abundance per species across trophic skew treatments in the presence and absence of predators. Values are means±1SE.

**Figure 4:**
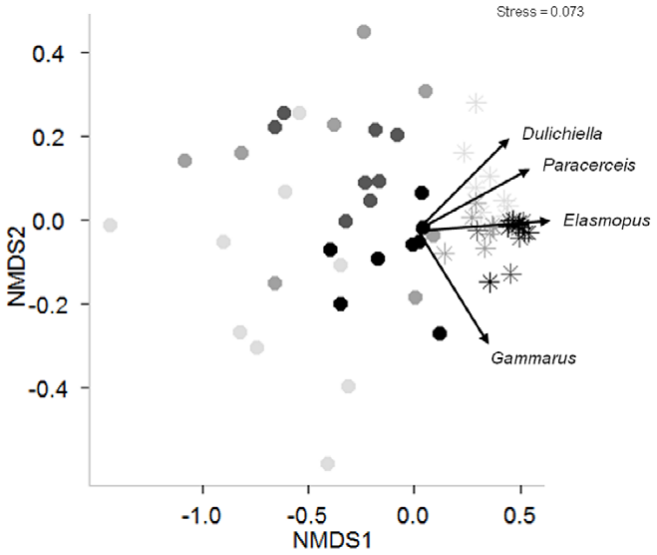
Non-metric multidimensional scaling scores for grazer community response to trophic skew and predator presence. Plot of the nonmetric multidimensional scaling (NMDS) scores for the two most important axes for all replicates using final grazer species composition data. Circles and stars represent the absence and presence of predators, respectively. Trophic skew treatments are represented from light to dark as follows: top-rich, neutral, slightly bottom-rich, and bottom-rich.

Incidental grazer immigration was minimal across all food web structures (∼39 individuals per algae-only control mesocosm, Fig. 2C). Both the presence of predators and changes in predator to prey richness influenced final grazer abundance (Table 1) and community composition (Fig. 3). The inclusion of predators reduced grazer abundance by 91%. Grazer abundance was greatest in the two bottom-rich food web structures regardless of the presence of predators (LSM contrast F_1,56_ = 27.53, P<0.0001, Fig. 2C) suggesting that macroalgal richness promoted grazer population growth. Grazer communities in treatments in which grazers were initially added (e.g., excluding algae-only treatments) were dominated by the amphipod *Elasmopus levis* (Fig. 3). Experimental grazer communities were significantly affected by predator presence (PERMANOVA, F_1,56_ = 97.032, P<0.001) and by the trophic skew treatment (F_3,56_ = 4.621, P = 0.006), as well as by the interaction between the two (F_3,56_ = 3.656, P = 0.009; Fig. 4). However, data failed the test of multivariate homogeneity (e.g., permutation analysis of betadisper results for homogeneity across trophic skew treatments, F_3,60_ = 2.804, P = 0.031) and treatment effects on grazer community composition should be interpreted cautiously.

## Discussion

Changing the structure of food webs, via reductions in length and diversity, can alter ecosystem functioning [39], [47]. The results of our experiment suggest that trophic skewing of species richness (changes in the ratio of predator to prey richness) can also affect ecosystem function in a diverse estuarine food web. Specifically, this trophic skewing of species richness can affect primary and secondary production as well as the strength of a trophic cascade.

Similar to results from other aquatic and terrestrial studies, we found that increasing primary producer richness increased production (primary and secondary biomass) after 24 days in our experimental communities [46], [48], [49], [50], [51]. The observed positive effects of increasing plant richness on plant biomass are thought to be largely due to complementarity and sampling effects, whereby the likelihood of incorporating facilitators and highly productive species, as well as heightening resource partitioning, increases with elevated richness [52].

Although increasing plant richness may increase plant biomass, these effects can be weak or undetectable in the presence of consumers due to grazing compensation [28], [53], [54], [55]. Here we found that increasing macroalgal richness from two to six species elevated macroalgal biomass (Fig. 2A), but this effect was not evident in the presence of grazers. This adds to a growing list of experimental manipulations demonstrating reduced diversity effects on production with the inclusion of a higher trophic level [35], i.e., under ecologically realistic conditions. However, it is unclear whether the added production is transferred up food chains resulting in greater production at top trophic levels [28].

Although a recent meta-analysis supports the concept that prey richness is a strong predictor of consumer effects and that more diverse prey communities can maintain higher abundances by containing species that are highly tolerant or resistant to consumers [56], we did not see a suppression of grazer effects on macroalgal biomass with increasing macroalgal diversity (Fig. 2A). Increasing macroalgal richness did not decrease the strength of top-down control by consumers [57], [58], [59]. It is possible, as with many short-term biodiversity studies in which plant biomass per species is initially low, that this experiment was not of sufficient duration to allow unpalatable algal species to replace the biomass of the more palatable species lost to herbivory [60]. Given that macroalgal diversity effects in this system are known to vary in space and time [61], the lack of environmental variation within the mesocosms may also have reduced our ability to detect macroalgal richness effects on primary production in the presence of grazers [62], [63]. Alternatively, as several of the macroalgal species in our species pool are known to be highly productive and readily consumed by these and other small crustacean grazers [45], [46], [64], [65], it is possible that increasing macroalgal richness increased overall macroalgal community palatability or the likelihood of incorporating a preferred macroalgal species (Table S1A), thereby promoting grazer populations which limited primary producer biomass. Elevating plant richness and the number of plant functional groups can promote herbivore diversity and overall herbivory [66], [67], potentially leading to the observed stronger top-down control in the most diverse macroalgal communities (Fig. 2A). This decoupling of plant richness and productivity in the presence of consumers may be a general trend in this and other systems with strong top down control [37], [68].

Generally, all grazer populations grew in response to the different experimental assemblages (Figs. 2C, 3). However, grazer abundance was greater in the more diverse macroalgal communities (Fig. 2C). Increased plant diversity can promote herbivore production directly through increased diet breadth (balanced diet hypothesis) and overall resource availability [28], [69], and indirectly by influencing herbivore-predator interactions through increased prey refuge availability and/or quality [70], [71]. Although studies in similar and other systems found that predators, while capable of destabilizing herbivore populations, had little effect on plants [44], [72], [73], here we found that predator presence can affect primary producers (Fig. 2). Elevating predator richness can promote primary producers by increasing prey capture – due to diet complementarity [45], [72], [74]–and prey antipredator behaviors such as reduced grazing, thereby releasing plants from herbivory and strengthening a trophic cascade [45], [75].

The effects of trophic skew on the strength of a trophic cascade in this model estuarine system, however, varied between the two primary producer groups (experimentally stocked macroalgae and naturally colonizing microalgae, Fig. 2D,E). Increasing prey richness relative to predator richness (e.g., from top-rich to bottom-rich food webs) strengthened the trophic cascade on macroalgae and generally promoted standing macroalgal biomass, but potentially weakened the trophic cascade on microalgae. A recent meta-analysis of work in detrital food webs, however, found that top-down effects of changing consumer (detritivore) richness had stronger effects on functioning (e.g., decomposition) than the bottom-up effects of changing resource (detritus) diversity [39]. Contrasts between this work in a ‘brown’ versus our study in a marine ‘green’ web may reflect inherent differences in processes associated with the two systems including the role of dynamic responses of ‘live’ resources to their consumers [35].

Increasing predator richness in our experimental communities was likely correlated with increased likelihood and potential promotion of omnivory (both a sampling and nonadditive richness effect), thereby promoting overall consumption of macroalgae and appearing to weaken the trophic cascade on these producers in top-rich food webs [45], [76]. Additionally, it is possible that the variable effects on producer functional type (experimentally stocked macroalgae versus the naturally colonizing microalgae) were due to changes in grazer foraging behavior. Grazer abundance increased in response to elevated plant richness (e.g., bottom-rich food webs), potentially increasing inter-and intraspecific competition and promoting grazer consumption of microalgae. Elevated predator richness in the top-rich food webs may have reduced grazer activity and increased use of macroalgal refuges such as *Dictyota menstrualis* and *Ulva lactuca* [71], [77], [78], reducing access to and overall consumption of microalgae in these treatments. Thus, trophic skew may affect different types and dynamics of primary producers, making it difficult to predict the overall effects of concurrent species gains and losses on primary production.

Community composition and identity effects may drive observed differences among experimentally skewed food webs. Elevated macroalgal richness, coupled with decreased predator richness, may have promoted grazer survivorship and population growth through increased refuge and food quality, and/or decreased predator efficiency of prey capture [45], [71]. An increased likelihood of incorporating unpalatable algae due to higher algal richness and a decreased likelihood of omnivory due to lower predator richness (Table S1A, B) could further promote algae in bottom-up skewed communities. Reduced predator richness in these food webs could also reduce predator efficiency if intraspecific competition among predators is stronger than interspecific interactions. As food webs become skewed, the identity of the species being gained or lost (e.g., whether they are an omnivorous predator or a palatable plant) as well as the trophic level in which they reside may become increasingly important.

Biodiversity can significantly affect primary production, nutrient cycling and community composition. Control of algal blooms, the yield of important commercial and recreational fisheries, and other ecosystem services may depend not only on the maintenance of biodiversity, but also on its distribution throughout a given food web. As substantial losses of species at local and global scales as well as local gains of non-native species are expected to continue [4], [5], [79], [80], understanding the effects of trophic skew on ecosystem functioning may be important for predicting the potentially synergistic effects of species extinctions and invasions on ecosystem functioning and will be an important challenge for empirical and applied endeavors across systems.

## Materials and Methods

### Mesocosms and Experimental Design

The experiment was performed in outdoor, flow through mesocosms at the University of North Carolina at Chapel Hill’s Institute of Marine Sciences (IMS) in Morehead City, NC in July 2007. We independently manipulated secondary consumer (referred to as predator throughout) and macroalgal richness to create four different food web structures (trophic skew treatment) mimicking different trophic skewing scenarios, with constant total community richness (Fig. 1A). Experimental food webs were skewed to be top-rich (2 macroalgal: 4 grazer: 6 predator species), neutral (4∶4∶4), slightly bottom-rich (5∶4∶3), or bottom-rich (6∶4∶2). Trophic skew treatments were crossed by three manipulations of food chain length (algae only, algae+grazers, algae+grazers+predators) to compare changes in trophic cascade strength across experimental communities (n = 9; 108 mesocosms total).

We used a substitutive design, manipulating initial algal and predator richness and identity while holding biomass and density constant per mesocosm (35 g algae and 6 predator individuals, *c*. 18 g) at densities comparable with natural levels in North Carolina subtidal communities [45], [81], [82], [83]. Algal and predator species composition per replicate were chosen randomly from a larger pool of nine macroalgae and eight predator species (Fig. 1B). Selection of species from these larger species pools allowed for a conservative test of richness effects in our system by varying community composition, but not richness, for replicates within a given treatment. This controlled for species identity and composition effects [84]. Algal composition varied among replicates and levels of trophic skew, but was replicated across the different food chain length treatments to account for the effects of initial algal composition on the final response variables. Initial grazer richness, composition and abundance were replicated such that each treatment received a mixture of four grazer species. We chose a substitutive design because it does not confound diversity with density, as do additive designs [76], [85]. Although replacement designs can potentially diminish intraspecific interactions by decreasing species-specific densities with increasing species richness [60], [86], they are useful in detecting complementarity effects [87].

Replicates were maintained in 30 L clear plastic aquaria provided with gravel-filtered seawater from Bogue Sound (see Fig. S1 for images of experimental mesocosms). Seawater flowed through 100 µm nylon mesh filter bags and was delivered through a dump bucket system that maintained aeration and approximated natural subtidal turbulence [45], [46], [88]. This system limited outside grazer recruitment, but not the passage of microscopic algal propagules that colonized the mesocosms (referred to throughout as “microalgae” as they were primarily composed of diatoms). Mesocosms were covered with 5 cm opening Vexar mesh lids and were placed in water tables to maintain constant temperature. Light, temperature and salinity within the mesocosms closely approximated field conditions in the nearby Bogue Sound during the course of the experiments [45], [89]. Mesocosms were rotated every 5 days to reduce positioning artifacts.

### Study System and Organisms

The South Atlantic Bight hard-substratum communities are highly diverse, composed of tropical and temperate species of algae, invertebrates and fishes [90]. Macro-and epiphytic algae, the main primary producers in this system, are intensely grazed by a diverse macroinvertebrate community composed largely of amphipods and isopods [88], [91], which are in turn consumed by an array of invertebrate and vertebrate predators including shrimp, crabs and fishes [81], [92]. Experimental communities featured local algal, grazer, and predator species that commonly co-occur and typically dominate hard-substratum sites of North Carolina estuaries. Organisms were collected and cultured or maintained in outdoor water tables at IMS prior to experimentation.

Chosen macroalgal species (Fig. 1B) are common in NC estuaries, although their abundances fluctuate seasonally [46], [90], [93]. We attached seven algal thalli haphazardly to 25×25 cm Vexar mesh screens (with 5 mm openings), which were secured to the bottom of each 30 L polypropylene mesocosm such that algae floated upright in natural orientation. Initial total algal biomass per mesocosm was held constant at 35 g with approximately 5 g per individual thalli attachment. Initial algal biomass was purposefully lower than field densities [46] in order to allow room for growth. Algal biomass was determined after first immersing the algae in seawater for at least 15 minutes and then spinning it 15 revolutions in a salad spinner to remove excess water. We dipped algae in a diluted pesticide, Sevin, and rinsed it with fresh seawater to remove existing mobile invertebrates and trace pesticides before placement in mesocosms [46], [88], [94]. Any fouling organisms (e.g., tunicates) were removed by hand prior to weighing; algal pieces with encrusting invertebrates (e.g., bryozoans) were replaced.

Mesocosms received an initial equal volume of grazers from a mixture of three amphipods (*Dulichiella appendiculata*, *Gammarus mucronatus*, and *Elasmopus levis)* and one isopod (*Paracerceis caudata*) prior to predator additions. These mesograzers are common in NC estuaries, achieving densities of 10–145 individuals g^−1^ wet mass of the alga *Sargassum filipendula* [95]. They also have short generation times, respond quickly to changes in habitat and predation, and consume various types of macro-and microalgae [81], [96], [97]. Each subsequent week an additional equal volume of grazers was added to each mesocosm to mimic natural recruitment [98] and to remove the possibility of predator overexploitation (for a total of *c*. 120 herbivores per mesocosm overall). Volume additions were subsampled (*n* = 20) and composed mostly of *E. levis* for the initial additions, and *D. appendiculata* and *P. caudata* for the recruitment additions. The initial volume addition was supplemented with five individuals of each grazer species to ensure that all replicates received the same grazer richness. Grazers were stocked within the lower range of natural field densities to allow for natural reproduction and population growth throughout the experiment [45], [99], [100].

Predator assignments were chosen randomly from a pool of functionally distinct invertebrates and fishes including omnivorous and strictly carnivorous species (Table 1). Due to low field abundances it was impossible to collect enough of any one of the mud and swimming crab species. To resolve this issue without risking elevating richness, each replicate assigned to either of these crab groups received individuals of only one species for that group throughout the duration of the experiment. Each mesocosm received six individual predators, which is within natural field densities [83]. Predators were collected within their respective average juvenile size classes. This was most important for *L. rhomboides*, which ontogenetically switches from a strict predator to an omnivore at 3.5 cm [101], [102], or around 2.5 g (feeding trial pilot study, *n* = 8). Total predator mass per mesocosm varied (0.29–3.71 g), but did not differ greatly among treatments. Mesocosms were checked daily and dead or stressed predators were replaced throughout the experiment. This predator press design maintained the potential for species interactions (e.g., intraguild predation), although it precluded direct, long-term effects of such encounters on lower trophic levels.

### Responses and Analyses

After 24 days we quantified treatment effects on grazer abundance, the biomass of all experimentally stocked macroalgae, and the accumulation of naturally colonizing algae (“microalgae”). This endpoint was based on observable changes in algal growth among treatments and represented approximately two overlapping grazer generations [103]. Grazers were preserved in 70% EtOH and later identified and counted. To assess microalgal production, we measured the chlorophyll *a* concentration from standardized 2 cm^2^ samples scraped from the side of each mesocosm. We extracted and quantified chlorophyll *a* concentration as in Bruno and O’Connor [45] to quantify microalgal accumulation. Trophic cascade strength was assessed as (R_p_–R_g_)/(R_a_–R_g_) where R is the macro-or microalgal response when in the presence of grazers _(g)_ and predators _(p)_, or the absence of both consumer groups_(a)_.

We used a linear model with the fixed effects of trophic skew (4 levels) and food chain length (3 levels) and their interaction, with initial algal composition type as a blocking factor using the procedure PROC MIXED in SAS (v. 2.11, Cary, NC) to analyze treatment effects on all response variables. Algal composition type had no effect and was subsequently removed from the model and the analyses re-run without this blocking factor [104]. Replicates were excluded from the analysis if the mesocosms cracked or received inadequate flow, or the predators exhibited chronic stress (13 mesocosms total). Algae-only replicates were excluded in the analysis of treatment effects on final grazer abundance as these mesocosms did not initially receive any grazers and total immigration was minimal (Fig. 3). Results for all analyses were transformed as necessary to meet the assumptions of normality and heteroscedacity [105].

Non-metric multidimensional scaling (NMDS) [106] conducted with the metaMDS function in the vegan package in R v. 2.14.0 [107] was used to graphically examine changes in grazer community composition across trophic skew treatments in the absence and presence of their predators. Bray-Curtis distance was used as it performs well in simulations for ecological data [108]. The results were plotted in 2-dimensions, and the envfit procedure in vegan [107] used to overlay species vectors. Replicate points which occur close together on the NMDS axes are similar in species composition. A permutational analysis of variance (PERMANOVA) using the adonis function in the vegan package was then used to test treatment effects on grazer community composition. Multivariate homogeneity of the treatments was assessed using the betadisper function in vegan, which is analogous to Levene’s test [109] for equality of variances.

## Supporting Information

Figure S1 **Experimental mesocosm setup.** Clockwise: mesocosms receiving filtered seawater, macroalgal community, and (courtesy of M. O’Connor) mesocosm side view.
(TIF)

Figure S2 **Images of final experimental algal communities.** Example algal communities after 24 days in experimental mesocosms exposed to different types and degrees of trophic skew. Images within the same column featured the same initial macroalgal community.
(TIF)

Table S1 **Experimental design.** Species composition of A) macroalgal and B) predator community in each mesocosm (experimental unit). Initial macroalgal biomass and predator abundance per species changed with species richness in a substitutive design. Abundance of predators by species is denoted in parentheses when multiple individuals were present. Macroalgae species in shaded cells are known to be chemically defended and less preferred by most of our experimental mesograzers, while species in lighter cells are increasingly palatable. Predator species in shaded cells are omnivorous, while species in light cells are strict carnivores.
(TIF)
