# Supplementary figures and images for: Effects of Trophic Skewing of Species Richness on Ecosystem Functioning in a Diverse Marine Community
Source: PLoS One. 2012 May 31;7(5):e36196. doi: 10.1371/journal.pone.0036196 (PMC3365057; doi:10.1371/journal.pone.0036196)

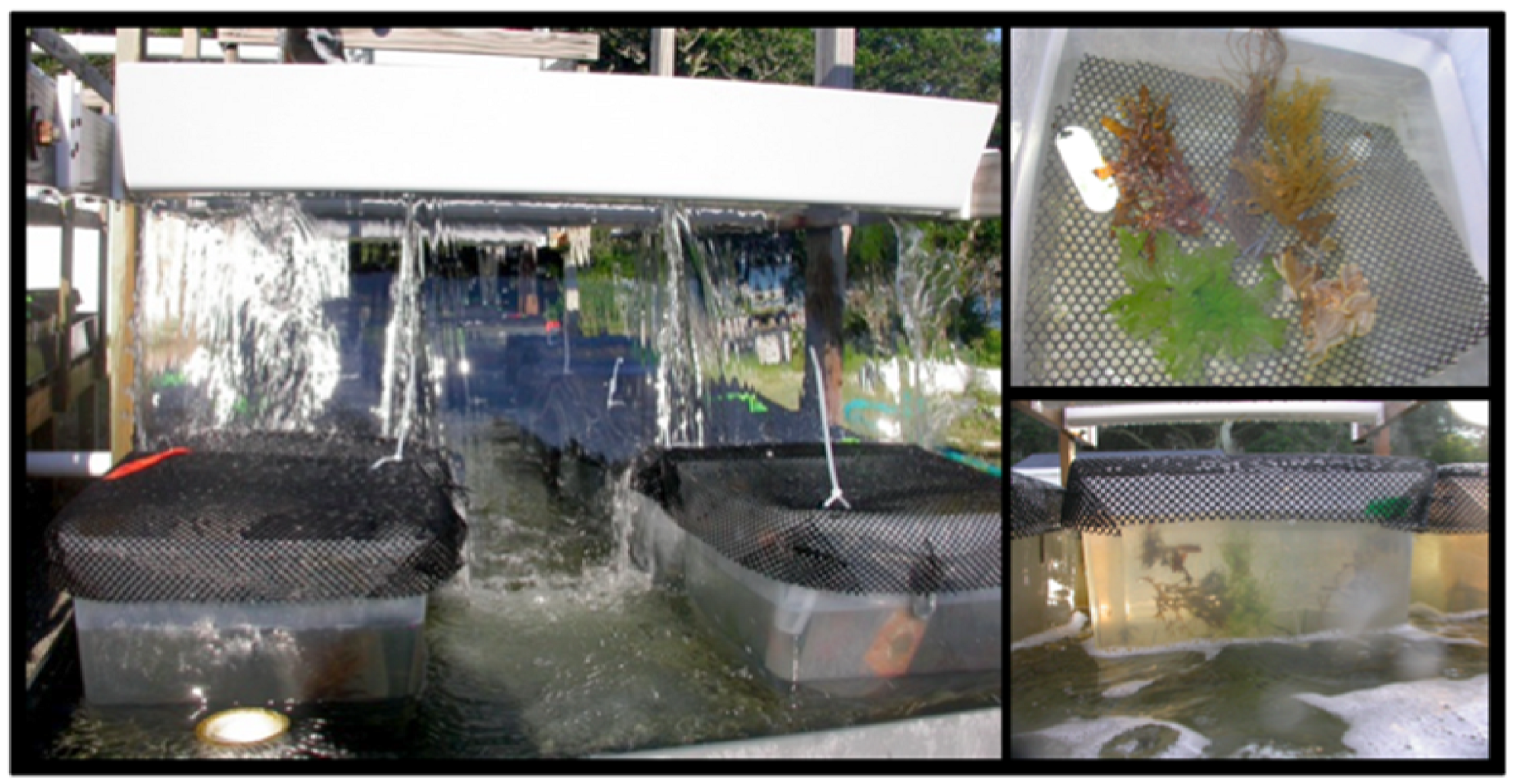

Supplement: Figure S1 — Experimental mesocosm setup. Clockwise: mesocosms receiving filtered seawater, macroalgal community, and (courtesy of M. O’Connor) mesocosm side view. (TIF) [file pone.0036196.s001.tif]

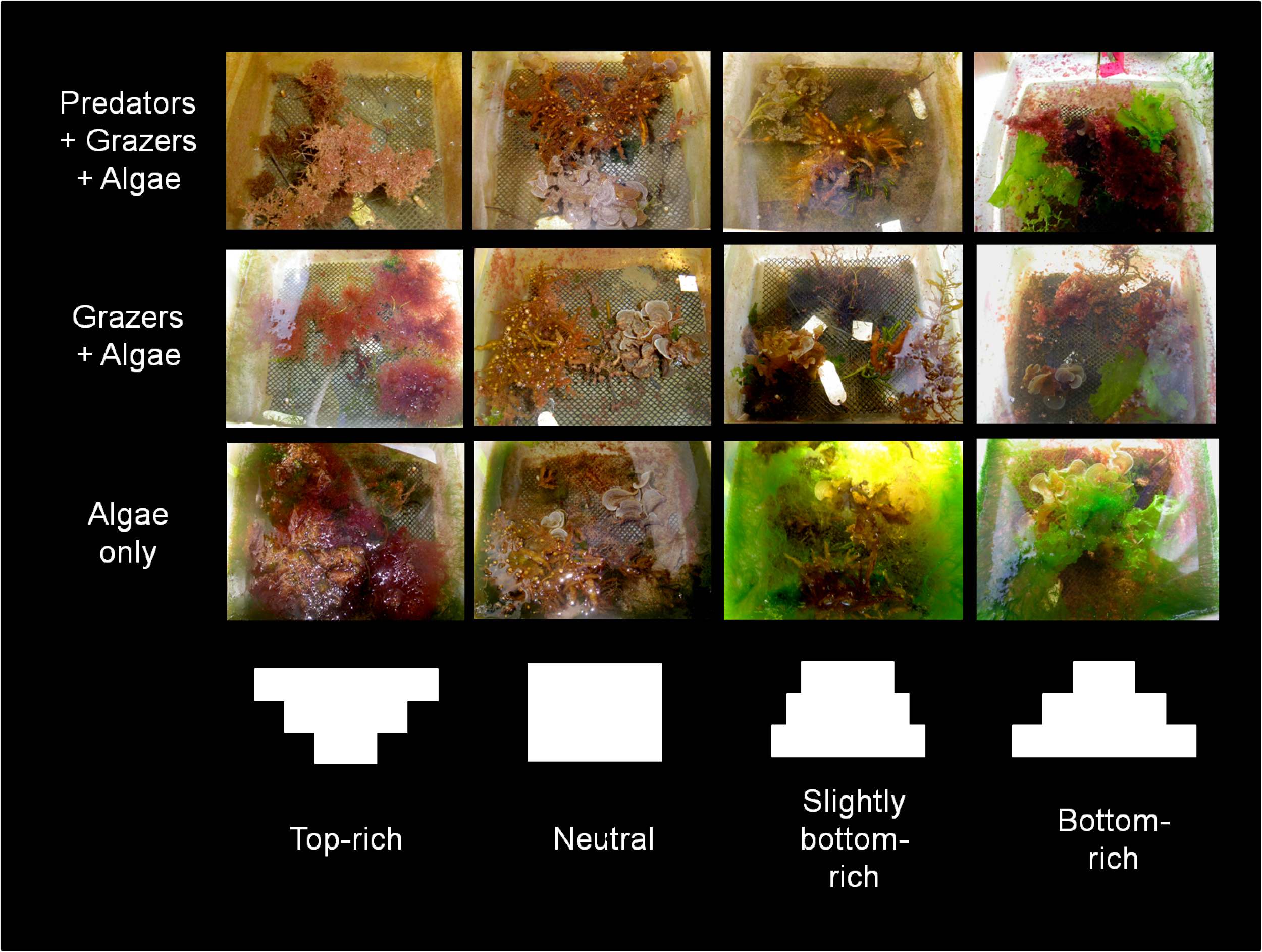

Supplement: Figure S2 — Images of final experimental algal communities. Example algal communities after 24 days in experimental mesocosms exposed to different types and degrees of trophic skew. Images within the same column featured the same initial macroalgal community. (TIF) [file pone.0036196.s002.tif]

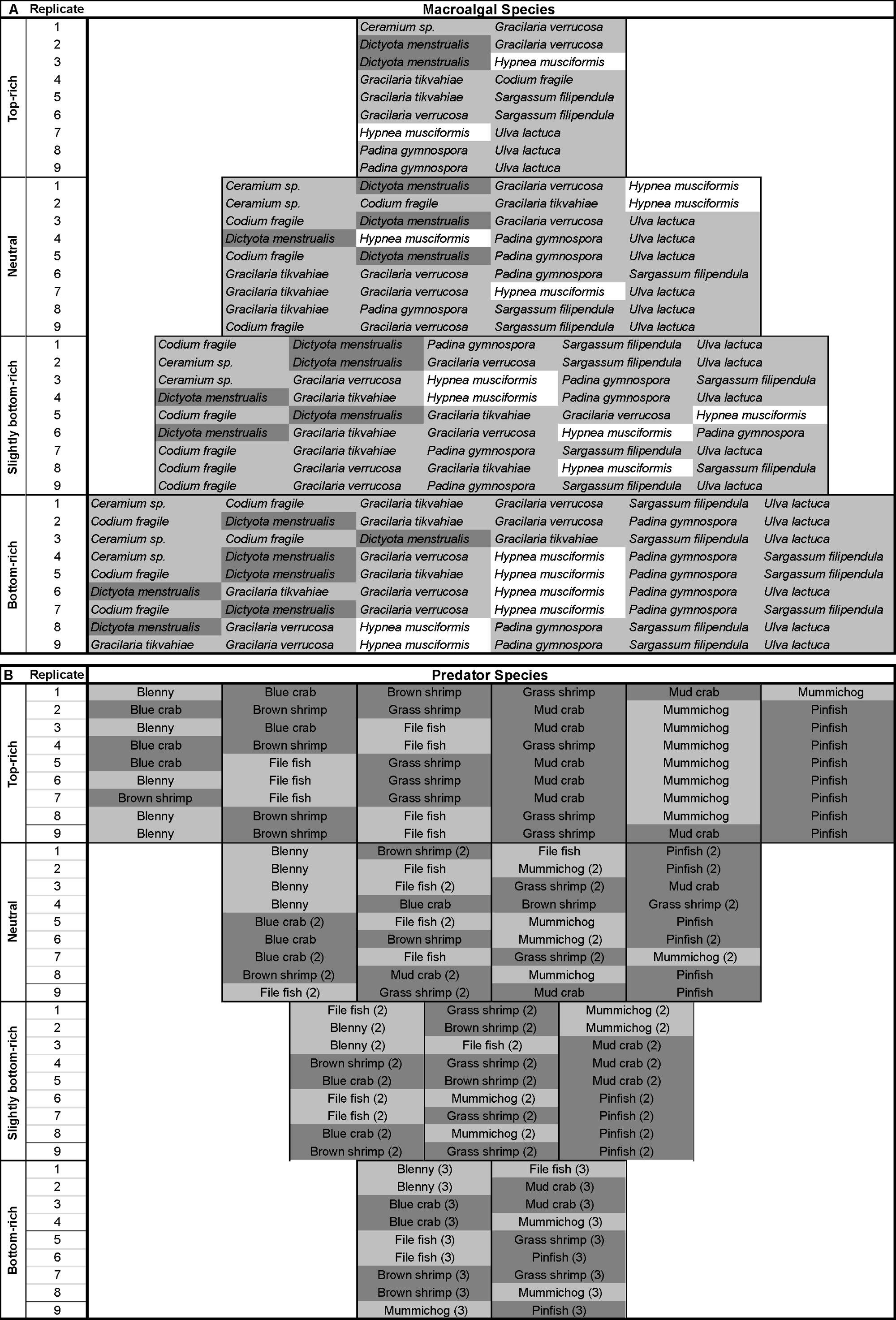

Supplement: Table S1 — Experimental design. Species composition of A) macroalgal and B) predator community in each mesocosm (experimental unit). Initial macroalgal biomass and predator abundance per species changed with species richness in a substitutive design. Abundance of predators by species is denoted in parentheses when multiple individuals were present. Macroalgae species in shaded cells are known to be chemically defended and less preferred by most of our experimental mesograzers, while species in lighter cells are increasingly palatable. Predator species in shaded cells are omnivorous, while species in light cells are strict carnivores. (TIF) [file pone.0036196.s003.tif]
